# Early and Delayed Alteration of Atrial Electrograms Around Single Radiofrequency Ablation Lesion

**DOI:** 10.3389/fcvm.2018.00190

**Published:** 2019-01-08

**Authors:** Stepan Havranek, Hana Alfredova, Zdenka Fingrova, Lucie Souckova, Dan Wichterle

**Affiliations:** ^1^Second Department of Medicine—Department of Cardiovascular Medicine, First Faculty of Medicine, Charles University and General University Hospital in Prague, Prague, Czechia; ^2^Department of Cardiology, Institute for Clinical and Experimental Medicine, Prague, Czechia

**Keywords:** radiofrequency catheter ablation, atrial fibrillation, pulmonary vein isolation, electrogram voltage, oedema

## Abstract

**Purpose:** The acute effect of radiofrequency (RF) ablation includes local necrosis and oedema. We investigated the spatiotemporal change of atrial electrograms in the area surrounding the site of single standardized pulse of RF energy.

**Methods:** The study enrolled 12 patients (45–67 years, 10 males) with paroxysmal atrial fibrillation (AF) undergoing ablation procedure with irrigated-tip ablation catheter and 3D navigation. The high-density mapping/remapping (129 ± 63 points) within the circular area with radius of ~10 mm, centered at the pre-specified posterior left pulmonary vein antrum ablation site was performed at baseline, immediately after single RF energy delivery (25 W, 30 s, 20 ml/min) and after 30 min waiting period. Bipolar voltages of atrial electrograms (A-EGM-biV) were averaged within the central and 12 adjacent left atrium segments and their relative change was studied.

**Results:** After the ablation, overall A-EGM-biV within the mapping zone (3.51 ± 1.89 mV at baseline) reduced to 2.83 ± 1.77 mV (immediately) and to 2.68 ± 1.58 mV (after 30 min waiting period). In per-segment pair-wise comparison, we observed highly significant change in A-EGM-biV that extended up to the distance of 8.8 mm from the lesion core. The maximum early A-EGM-biV attenuation by 39–49% (*P* < 0.001) was registered in segments adjacent to pulmonary vein ostia. The subsequent (delayed) A-EGM-biV reduction by 17–24% (*P* < 0.05) was observed in opposite direction from the lesion center.

**Conclusions:** Significant alteration of atrial electrograms was detectable rather distant from the central lesion. Spatiotemporal development of ablation lesion was eccentric/asymmetric. While acute A-EGM-biV reduction can be attributed predominantly to direct thermal injury, delayed effects are probably due to oedema progression.

## Introduction

Pulmonary vein (PV) isolation (PVI) for atrial fibrillation (AF) is an established therapy in selected patients (1). Nevertheless, resumption of PV-left atrial (LA) conduction is exceedingly common and thought to be responsible for the vast majority of post-ablation atrial tachyarrhythmia recurrences (2).

It is well-known, that the acute effect of radiofrequency (RF) ablation consists of local necrosis, collateral oedema, and atrial stunning (3, 4). In the absence of cellular death, reversible tissue oedema or thermal stunning may account for the discordance between the incidence of acute and chronic PVI (5–7), because injured, but still viable, myocardium may eventually recover its conduction properties and result in late PV reconnection (8). Furthermore, the phenomenon of oedema may protect the ablative energy from reaching the deeper layers of the myocardium and could be one of the reasons for the difficulty in creating conduction block.

Inspection of atrial electrogram (A-EGM) morphological/voltage change during ablation is well-recognized method for the assessment of lesion efficacy. However, conduction gaps are frequently located in epicardial layers of atrial myocardium. The larger distance from endocardially-positioned catheter bipole may be also augmented by the oedema-induced thickening of the atrial wall Therefore, gap-related electrical activities might be difficult to identify because of far-field morphology of corresponding A-EGMs (9).

Understanding the factors that influence A-EGM voltages might improve ablation effectiveness and clinical outcome. However, data on the immediate effect of RF lesion on A-EGM voltages are lacking. The aim of this prospective study was to investigate the spatiotemporal change of A-EGM in the area surrounding the site of single standardized pulse of RF energy.

## Methods

The study was approved by the local ethics committee and all patients signed an informed consent with the procedure. All antiarrhythmic drugs except amiodarone were discontinued for at least five half-lives prior to the study. Consecutive patients undergoing PVI for paroxysmal AF without structural heart disease who had no low voltage areas identified at the posterior LA wall (bipolar voltage <0.5 mV) during point-by-point electroanatomic mapping and who were in sinus rhythm at the beginning of the study protocol were enrolled.

### Ablation Procedure

The catheter ablation was performed in fasting state under the mild analgosedation with midazolam and fentanyl. It was guided by electroanatomic mapping using a CARTO^TM^ system (Biosense-Webster, Diamond Bar, CA, USA) and intracardiac echocardiography (AcuNav^TM^ catheter, Siemens, Germany) according to local standards. Briefly, after double transseptal puncture, the single 20-polar circular catheter (Lasso^TM^, Biosense-Webster, Diamond Bar, CA, USA) were placed at the ostia of PVs to record PV potentials. An LA reconstruction was achieved by point-by-point electroanatomic mapping with an open irrigation 3.5-mm-tip ablation catheter (NaviStar Thermocool, Biosense-Webster) during sinus rhythm. Tagging of PV ostia on the electroanatomic map was facilitated by intracardiac echocardiography. Radiofrequency (RF) energy was delivered (EP Shuttle^TM^, Stockert, Freiburg, Germany) for 30 s in the power-control mode (20–30 W) with irrigation flow rate of 20 ml/min (Cool Flow^TM^ pump, Biosense-Webster) and the temperature limit of <42°C. The energy was reduced when ablated in posterior antrum of PVs. Intravenous heparin was administered to maintain an activated clotting time of >300 s throughout the procedure. Single circumferential set of RF lesions was used to isolate ipsilateral PVs. The end-point of the PVI was defined as the disappearance or dissociation of PV potentials as well as the loss of capture of the LA by circumferential pacing from all PVs.

### Study Protocol

**Figure 1:**
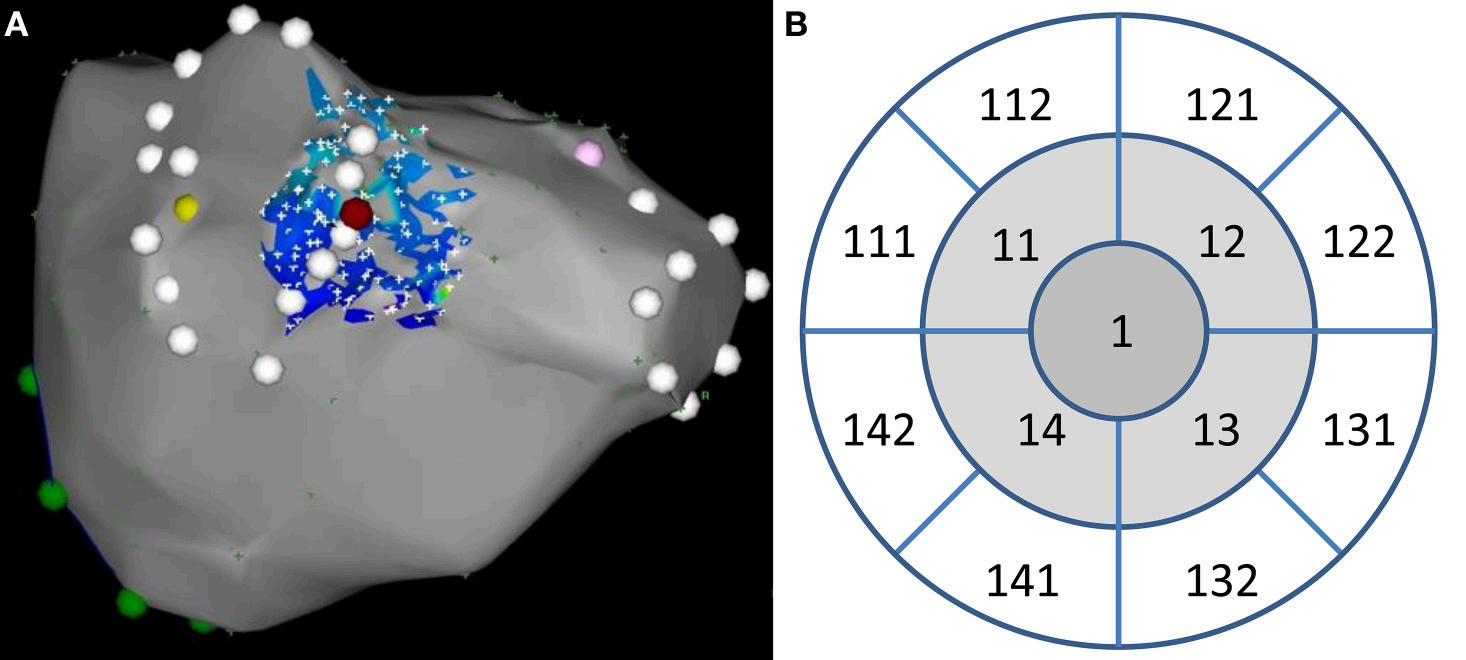
**(A)** An example of high-density bipolar voltage remap (low-color and high fill-threshold setting) at baseline. The site of index ablation is depicted by purple tag (posteroanterior view). PV antra are tagged by white points. **(B)** Corresponding layout of 12 segments used for electrogram analysis. Ablation site belongs to segment #1.

Patients without stable sinus rhythm were excluded. After completing the electroanatomic map of LA, the optimum site for “study” ablation was found and tagged at the smooth posterior LA wall at the intended wide circumferential isolation line. The catheter stability with minimum sliding was verified by intracardiac echocardiography and stable A-EGM morphology. The site of “study” ablation was also selected to allow comfortable catheter navigation and mapping in its neighborhood in order to prevent mechanical irritation with excessive production of atrial premature complexes. High-density voltage remapping within the circular area with radius of ~10 mm, centered at the tagged point was performed (Map 1). Single pulse of RF energy (25 W/30 s/irrigation of 20 ml/min) was delivered to the pre-specified target site under the control of catheter stability as described above (Figure 1A). The high-density remapping within the same area was performed immediately after the RF energy delivery (Map 2) and repeated after 30 min waiting period (Map 3). During this period the right-sided PV were isolated. Finally, isolation of left-sided PVs was finished.

### Data Processing

All three high-density remaps were meticulously inspected and manually edited to exclude all artifacts and cardiac cycles of non-sinus origin. Three-dimensional coordinates and corresponding bipolar voltages (A-EGM-biV) at all remapping points were exported and processed by purpose-written software. Specifically, points were projected to optimum common “en face” plane with ablation spot in the center. They were subsequently assigned to the central or one of 12 adjacent LA segments and their A-EGM-biV were averaged for each segment. The central segment had radius of 3 mm (area 28.3 squared mm). The external radii of adjacent segments were set to 6.7 and 10.8 mm for middle and outer segments, respectively, to get identical area for all segments (Figure 1B). Consequently, the mean distances of middle and outer segments from the lesion core was 4.9 and 8.8 mm, respectively.

### Statistical Analysis

Continuous variables were expressed as means with standard deviations and compared by the two-tailed *t*-test for dependent or independent samples, as appropriate. Not normally distributed variables were expressed as medians and interquartile range (IQR) and compared by Mann-Whitney *U*-test or Wilcoxon matched-pairs test, as appropriate. A *P* < 0.05 was considered significant. All analyses were performed using STATISTICA version 12 software (Statsoft, Inc., Tulsa, USA).

## Results

Out of 17 enrolled patients, two patients were excluded because of presence of low-voltage areas at the posterior LA wall and three patients were excluded because of AF onset during the study protocol. A total of 12 patients (10 males) with median age of 61 (IQR: 54–67) years were finally analyzed. Their baseline clinical and procedural characteristics are shown in Table 1.

**Table 1:** Baseline demographical, clinical, and procedure characteristics.

**N**	**12**
Males	10 (83%)
Age (years)	61 (45–67)
Arterial hypertension	8 (67%)
Diabetes mellitus	1 (8.3%)
Coronary artery disease	1 (8.3%)
Indexed LA volume estimated by ECHO (ml/m^2^)	36 (32–36)
Left ventricular ejection fraction (%)	61 (62–65)
LA volume estimated by 3D electro-anatomical mapping (ml)	118 (102–144)
Amiodarone	1 (8.3%)
Mapping points—dense map # 1	113 (54–167)
Mapping points—dense map # 2	114 (69–160)
Mapping points—dense map # 3	151 (110–191)

*Values are in format median (interquartile range) or n (%). LA, left atrium*.

Overall (mean of per-segment means) A-EGM-biV was 2.39 ± 1.34 mV at baseline. A-EGM-biV in individual segments at baseline, immediately after ablation and after 30 min are depicted in Table 2. Overall A-EGM-biV was significantly lower (1.76 ± 1.05 mV, *p* < 0.0001) in segments adjacent to PV ostia (# 11, 111, 112, 14, 141, and 142) compared to outer segments (2.69 ± 1.49 mV).

**Table 2:** Absolute values of bipolar atrial electrogram voltages in individual segments at baseline and two remapping periods.

	**Map 1 (baseline)**	**Map 2 (after 5 min)**	**Map 3 (after 30 min)**
1	2.33 ± 1.09	1.36 ± 0.66	1.08 ± 0.57
11	1.99 ± 1.12	0.87 ± 0.40	0.93 ± 0.46
12	2.79 ± 1.13	2.10 ± 1.19	1.74 ± 0.91
13	3.14 ± 1.46	2.49 ± 1.13	2.02 ± 1.00
14	1.95 ± 0.85	1.04 ± 0.46	0.91 ± 0.50
111	1.34 ± 0.89	0.91 ± 0.66	0.90 ± 0.73
112	1.83 ± 1.01	1.18 ± 0.78	1.30 ± 0.90
121	2.29 ± 1.46	1.95 ± 1.02	2.20 ± 0.80
122	3.50 ± 1.75	3.17 ± 1.57	2.94 ± 1.56
131	3.26 ± 1.61	3.77 ± 1.53	3.04 ± 1.58
132	3.01 ± 1.26	3.06 ± 1.31	2.36 ± 1.10
141	2.15 ± 0.91	1.50 ± 0.79	1.35 ± 0.69
142	1.60 ± 0.94	0.92 ± 0.51	0.92 ± 0.54

*Values are in format mean ± SD. Localization of segment: see Figure 1. Map 1: baseline, Map 2: <5 min after index ablation, Map 3: >30 min after index ablation*.

**Figure 2:**
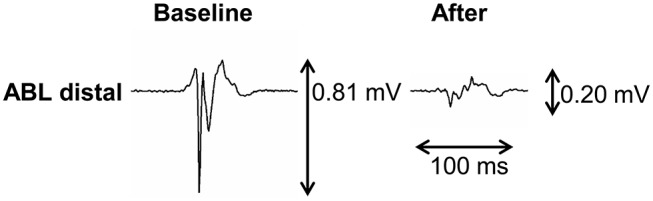
An example of changed bipolar atrial electrogram voltage after ablation.

Representative example of A-EGM change exactly at the site of ablation is shown in Figure 2. In the whole mapping zone, we observed significant reduction in overall A-EGM-biV from 2.39 ± 1.34 mV to 1.84 ± 1.34 mV immediately after ablation and 1.66 ± 1.17 mV after 30 min waiting period (both *P* < 0.01).

**Figure 3:**
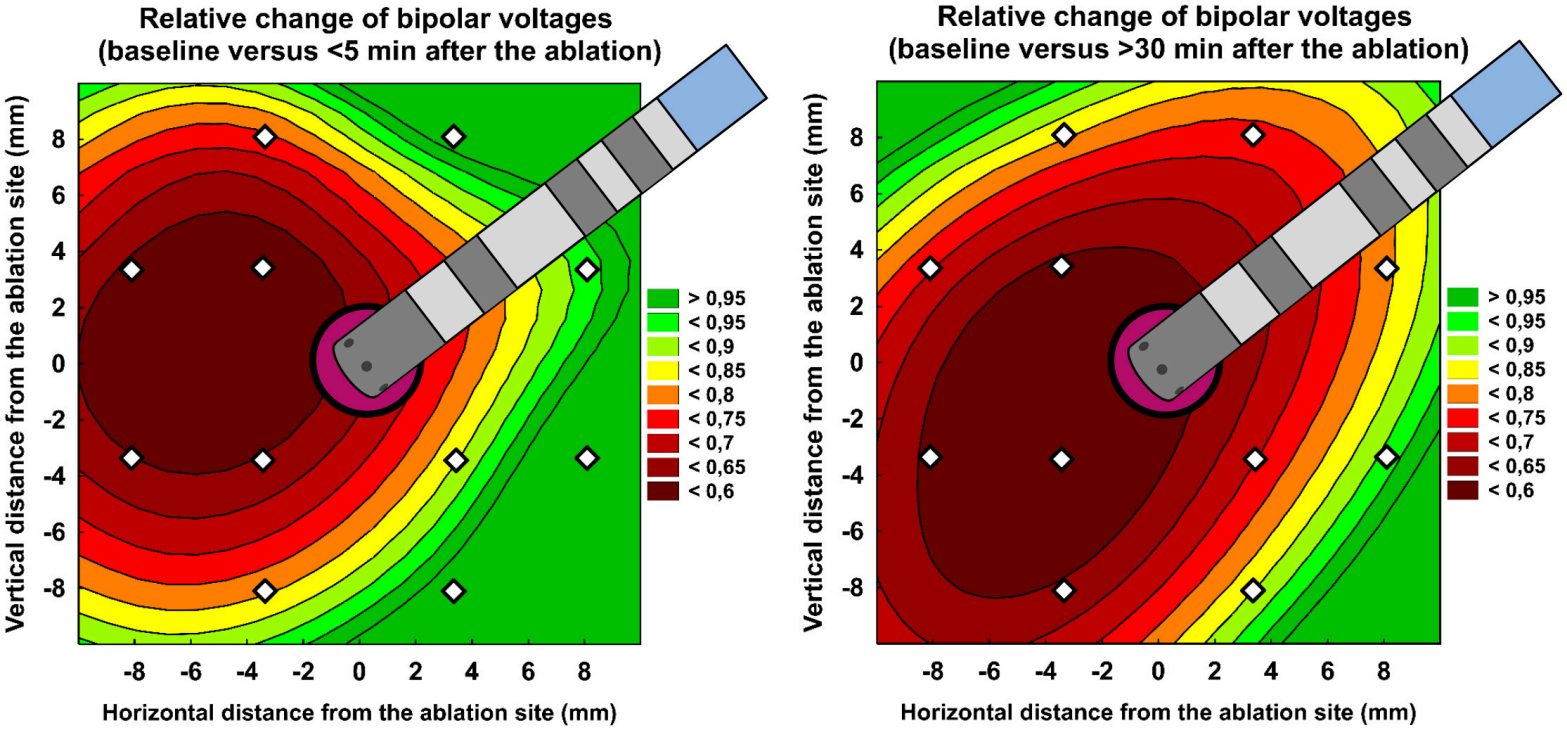
Relative change of bipolar atrial electrogram voltages: < 5 min after ablation vs. baseline (left panel) and >30 min after ablation vs. baseline (right panel). Color scale represents the intensity of relative change. The central purple circle corresponds to ablation core. White dots annotate centres of each analyzed segment. See Figure 1 for segment layout. The central purple tag corresponds to ablation site. Ablation catheter is displayed in proportion to the figure scale in millimeters.

**Figure 4:**
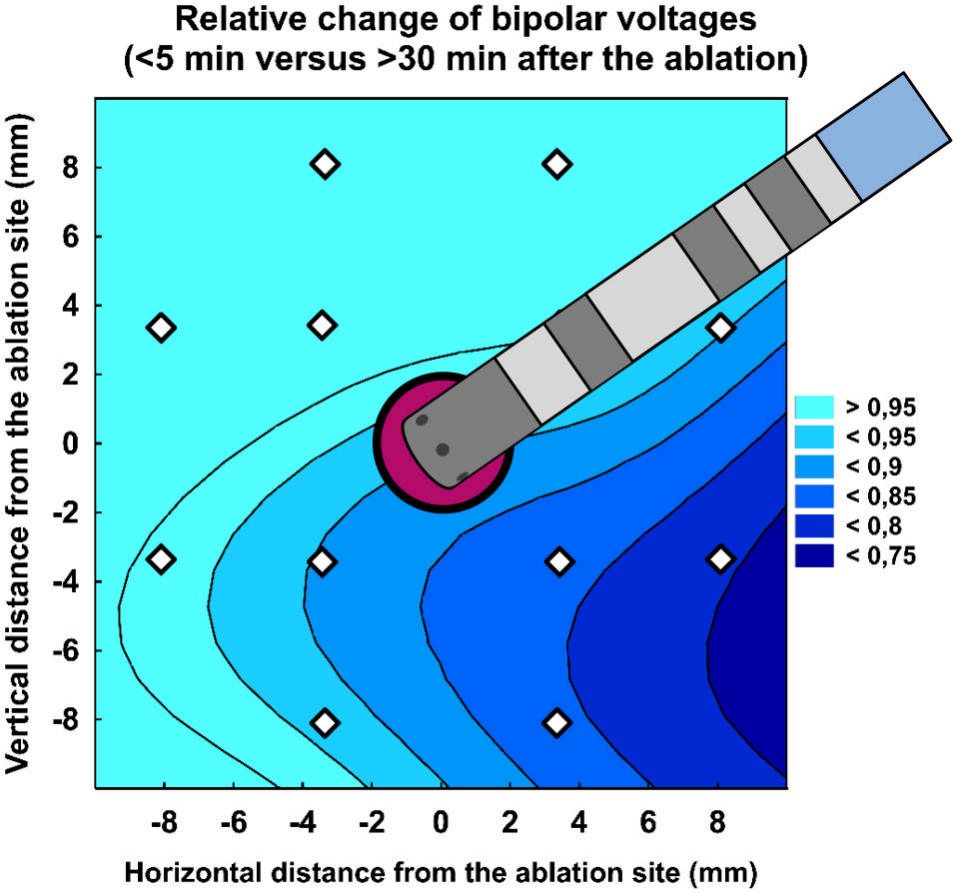
Relative change of bipolar atrial electrogram voltages (>30 min vs. < 5 min after the ablation). Color scale represents the relative change.

Table 3 shows per-segment pair-wise comparison of relative changes of A-EGM-biV between all three high-density maps. With central segment excluded, the maximum early A-EGM-biV attenuation (Map 2–Map 1) up to −49% (*P* < 0.001) was observed in segments adjacent to pulmonary vein ostia (Figure 3). The delayed A-EGM-biV reduction (Map 3–Map 2) up to −24% (*p* < 0.05) was most prominent in opposite direction from the lesion center (Figure 4).

**Table 3:** Relative change in bipolar atrial electrogram voltages in individual segments between consecutive maps.

**Segment**	**Map 2/1**	***p*^**1**^**	**Map 3/1**	***p*^**2**^**	**Map 3/2**	***p*^**3**^**
1	0.66 ± 0.24	0.0008	0.60 ± 0.38	0.006	0.86 ± 0.35	0.21
11	0.51 ± 0.26	0.0003	0.54 ± 0.23	0.0002	1.09 ± 0.26	0.25
12	0.71 ± 0.27	0.005	0.64 ± 0.26	0.001	1.10 ± 1.04	0.75
13	0.84 ± 0.36	0.16	0.70 ± 0.33	0.009	0.83 ± 0.18	0.007
14	0.62 ± 0.28	0.001	0.52 ± 0.24	0.00005	0.87 ± 0.33	0.21
111	0.60 ± 0.20	0.0001	0.70 ± 0.39	0.04	1.19 ± 0.69	0.39
112	0.71 ± 0.23	0.003	0.83 ± 0.32	0.12	1.32 ± 0.67	0.15
121	1.12 ± 1.05	0.77	0.73 ± 0.29	0.047	1.13 ± 0.36	0.35
122	0.86 ± 0.20	0.07	0.79 ± 0.19	0.01	0.90 ± 0.15	0.06
131	1.24 ± 0.32	0.045	0.92 ± 0.39	0.49	0.76 ± 0.27	0.01
132	1.05 ± 0.23	0.46	0.85 ± 0.31	0.13	0.81 ± 0.25	0.03
141	0.78 ± 0.59	0.25	0.61 ± 0.22	0.0001	0.95 ± 0.33	0.58
142	0.61 ± 0.27	0.0007	0.60 ± 0.25	0.0002	0.98 ± 0.34	0.84

*Values are in format mean ± SD. p^1^–comparison of Map 2 and 1; p^2^–Map 3 and 1; p^3^–Map 3 and 2, respectively. Localization of segment: see Figure 1. Map 1: baseline, Map 2: <5 min after index ablation, Map 3: >30 min after index ablation*.

## Discussion

The major finding of the study is that spatiotemporal development of single ablation lesion in PV antrum was eccentric/asymmetric with prominent initial A-EGM voltage reduction in regions adjacent to PV ostia and delayed A-EGM voltage decrease expanded in opposite direction. With respect to time-specific changes of ablation lesion, we hypothesize that detected delayed bipolar A-EGM voltages reduction, especially at regions adjacent to posterior wall and inferior segments, is due to tissue swelling.

It is well known that RF energy causes resistive heating of the tissue in the proximity of ablation catheter tip, followed by conductive heating of surrounding tissue (3). As the radial distribution of tissue temperature follows an inverse proportion with increasing distance from the catheter tip, an area with sublethal temperature surrounds the zone of permanent damage (3). The pathological studies have shown that acute lesion created by RF current consists of a central zone of coagulation necrosis surrounded by a zone of hemorrhage and inflammation followed by oedema (10–14). The existence of surrounding oedema after ablation was confirmed in different imaging studies based on echocardiography (13, 15–22), cardiac MRI (14, 23–25), electron beam tomography (22) or ultrasound-based shear elastography (26). Pre-clinical pathologic and ultrasound data suggest that RF ablation-mediated cardiomyocyte and interstitial oedema occur almost immediately after energy delivery, and extends beyond the point of ablation (13). In another echocardiographical case series, the early appearance (within 15 s) of oedema during energy delivery was observed (17). In MRI study, Lardo et al. detected that lesions reached maximum size 5 min after ablation, whereas lesion signal intensity increased linearly with time but then reached a plateau at 12 min, which seems to be consistent with the temporal physiology of local acute interstitial oedema (14). Ultrasound-based elastography imaging techniques detected an immediate increase in tissue stiffness during RF delivery. The relative stiffness in the region adjacent to the area of stiffness increased slightly during the first 15 min, consistent with local fluid displacement (26).

In the context of lesion physiology, an acute A-EGM-biV reduction immediately after ablation in central zone close to ablation core corresponds most likely to direct thermal injury. However, since immediate oedema formation during RF delivery was observed in the real-time ultrasound study (27), its participation in early attenuation of A-EGM cannot be ruled out. Interestingly, the voltage reduction after ablation has asymmetrical distribution with larger voltage reduction in segments adjacent to PV ostia. We propose three possible explanations of this phenomena. First, the myocardium sleeves covering the PVs become gradually thinner toward the pulmonary segment of the vein and ended distinctly (28). Relatively deeper energy penetration may be expected in thinner myocardium in the direction to PV ostia, despite increasing distance from the ablation electrode. Second, single ablation in PV antrum leads to loss of direct electrical continuity resulting in change of direction of impulse propagation in the segments between the RF lesion and PV ostia. Consequently, propagation of the waveform that is more perpendicular to the mapping catheter bipole can be associated with A-EGM-biV reduction. Third, the geometrical center of recorded A-EGM is in the middle between centers of two distal electrodes of mapping catheter, i.e., ~3 mm from the catheter tip and might not precisely match the sensor position. When catheter is positioned parallel to atrial wall during mapping of posterior left PV antra, such a mismatch may be responsible for biased projection of local electrograms relative to the anatomical tag of ablation site.

The finding of temporal and spatial A-EGM-biV reduction is not a fully novel observation. It is well known, that PVI leads to the injury of myocardial tissue adjacent to antrum, which may contribute to the reduction of atrial voltage after PVI. The absence of voltage reduction in the atrial wall 1 cm from the PV lesions at the end of PVI was related to recurrent AF (29). It has also been observed, that the oedematous changes in the LA wall often extended to regions remote from the PVs, where the RF ablation was not applied. Duytschaever et al. reported an electrogram voltage reduction at the LA posterior wall not only within but also outside the encircled PVs after circumferential PVI. The authors suggested that an electrogram voltage reduction at the LA posterior wall remote from the ablated PVs, probably owing to oedematous changes, may have contributed to the modification of the arrhythmia substrate (30). The correspondence between acute oedema on MRI to regions with reduced A-EGM-biV < 0.05 mV has been also shown (25).

In our study, standard catheters with relatively large distal bipole were used that record a combination of local signals and far field activity. The far-field component can be attenuated by the use of catheters with mini-electrode bipoles generating “ultra–local” tissue intracardiac electrograms. Lloyd et al. have shown, that A-EGMs characteristics from miniature embedded electrodes had greater voltages at non-ablated sites and lower voltages at ablated sites than standardly obtained A-EGMs (31). It seems that catheters with mini electrodes bring about additional benefit in distinguishing of ablated from non-ablated tissue in parallel with high spatial resolution (31). The results were confirmed by one recent *in silico* study (32). It is plausible to hypothesize that our results (including delayed changes) could have been more prominent if A-EGMs from miniature embedded electrodes had been analyzed. However, such catheters are not established in routine AF management and our results correlate better with phenomena that may be observed in clinical practice.

The bipolar voltage might be also influenced by catheter orientation and contact force at catheter-tissue interface (33).

Despite the angulation of catheter was not exactly controlled in our study, the same transseptal sheath was used and its position was verified by fluoroscopy. We believe that individual inaccuracies were mutually nullified when high-density mapping and per-segment averaging was used. All three investigational maps were created in the same fashion, so that any systematic error was not likely involved.

It is not clear how the use of contact force sensing catheter would affect the results. Several studies did not demonstrate consistent correlation between contact force and A-EGM-biV (34–36). In our study all mapping points were manually inspected and all premature atrial beats, noisy signals and any suspected far-field EGMs were excluded from the analysis.

### Limitations

Apart from above discussed points, the study has several other limitations. First, respiratory movements could also interfere with spatial accuracy of mapping points, although the respiratory variations are much less pronounced at posterior wall compared to other LA regions. Software option for respiratory compensation was disabled because this would prevent to acquire sufficient number of mapping points in limited time. Second, we cannot exclude that properties of myocardium were changed by mechanical trauma due to the repeated contact mapping. Third, the study investigated conventional RF delivery and the obtained results cannot be extrapolated to other RF ablation modes, e.g., those with high-power short-duration setting. Finally, it remains unknown whether observed changes at the posterior antrum of left PVs could be comparably reproduced in other LA regions with much thicker myocardium, more complicated anatomy or presence of significant scarring.

## Conclusions

Significant alteration of atrial electrograms was detectable rather distant from the center of RF ablation lesion. Spatiotemporal development of ablation lesion was eccentric/asymmetric. While acute A-EGM voltage reduction can be attributed predominantly to direct thermal injury, delayed effects are probably due to oedema progression.

## Ethics Statement

This study was carried out in accordance with the recommendations of Ethics committee of the General University Hospital in Prague with written informed consent from all subjects. All subjects gave written informed consent in accordance with the Declaration of Helsinki. The protocol was approved by the Ethics committee of the General University Hospital in Prague.

## Author Contributions

SH participated on data collection and analysis and drafted the manuscript. HA, ZF, and LS participated on data collection and analysis. DW conceived the study design, participated on data collection and analysis, performed statistical analysis, and critically revised the manuscript for important intellectual content. All authors read and approved the final manuscript.

### Conflict of Interest Statement

The authors declare that the research was conducted in the absence of any commercial or financial relationships that could be construed as a potential conflict of interest.
